# Supplementary material for: Subcellular distribution of ezrin/radixin/moesin and their roles in the cell surface localization and transport function of P-glycoprotein in human colon adenocarcinoma LS180 cells
Source: PLoS One. 2021 May 11;16(5):e0250889. doi: 10.1371/journal.pone.0250889 (PMC8112653; doi:10.1371/journal.pone.0250889)

Supplemental materials and methods

Rho123 accumulation study

LS180 cells were seeded at a density of 1.0×10^5 cells/dish in polylysine-coated 35-mm glass bottom dish with 14-mm inner diameter (Matsunami Glass, Osaka, Japan) and were incubated overnight at 37°C in a humidified atmosphere with 5% CO_2 to allow for attachment. Then cells were pretreated with 5, 10 or 50 μM of verapamil (FUJIFILM Wako Pure Chemical, Osaka, Japan), a classical inhibitor of the P-gp function, for 30 min followed by incubation with 10 μM of Rhodamine123 (Merck, Darmstadt, Germany) as a P-gp substrate in the dark for 2 h at 37°C in a humidified atmosphere with 5% CO_2. After washing the cells with D-PBS for three times, photomicrographs were taken at 0.5–1.0-μm intervals for z-axis at an original magnification of 20× with a Nikon A1 confocal laser microscope system (Nikon Instruments, Tokyo, Japan). The two- or three-dimensional images were reconstructed from the obtained pictures using the NIS-Elements Ar Analysis software (Nikon Instruments).

Supplemental figure

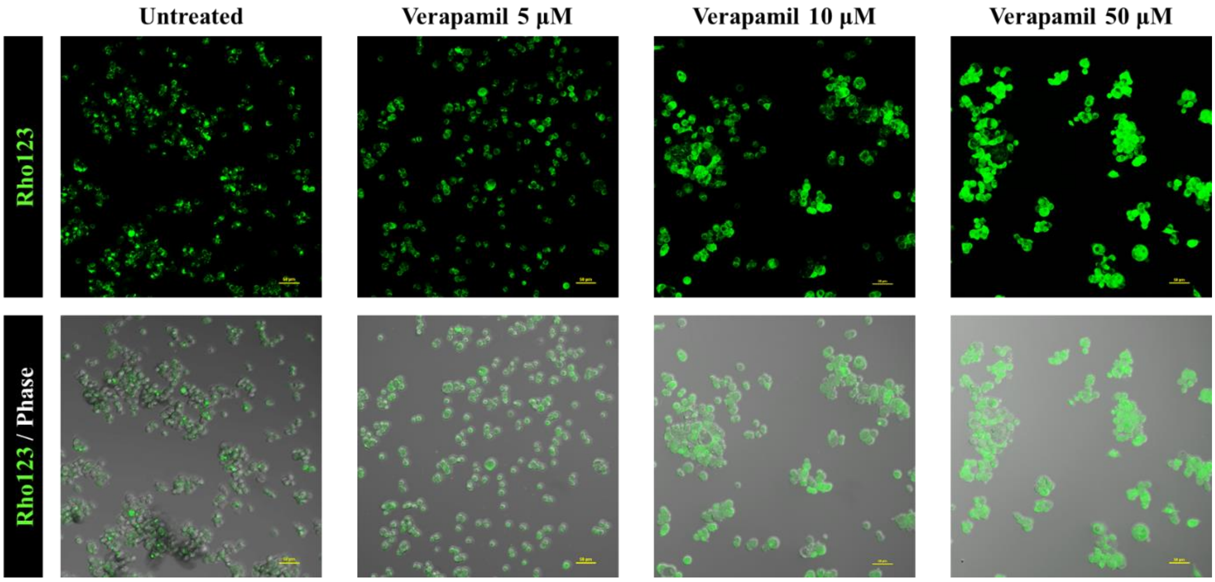

Supplement: S1 Fig — (PDF) [file pone.0250889.s001.pdf]
