# Supplementary material for: Subcellular distribution of ezrin/radixin/moesin and their roles in the cell surface localization and transport function of P-glycoprotein in human colon adenocarcinoma LS180 cells
Source: PLoS One. 2021 May 11;16(5):e0250889. doi: 10.1371/journal.pone.0250889 (PMC8112653; doi:10.1371/journal.pone.0250889)
Supplement: Attachment — Submitted filename: Response to reviewers_Kobori et al..docx [file pone.0250889.s002.docx]

**Reply comments to #Reviewer 1**

We would like to thank #Reviewer 1 for valuable comments and suggestions on our manuscript. We have carefully read all of your comments and suggestions and have made the corrections in the revised version of manuscript. Detailed responses to your comments are listed below, and we highlighted all changes with word track changes in the file labeled ‘Revised Manuscript with Track Changes’. We hope this revised manuscript would be satisfactory for publication in *PLoS One*.

***Comment 1. The authors should test the involvement of both P-gp and the ERM-mediated membrane-to-cytoskeleton connection at different pH, in order to render their findings more real.***

**Reply Comments.**

We would like to appreciate #Reviewer 1’s valuable suggestion. As #Reviewer 1 pointed out, we performed additional study to investigate whether ERM proteins also regulate the plasma membrane localization of P-gp under the condition at the acidic pH of tumor microenvironment. Based on the results of additional experiments, we added following sentence in the Materials and Methods, Results, Discussion, and References to the revised version of manuscript.

**Materials and Methods (Line 68 – 73)**

The acidic cell culture medium (pH 6.5) was obtained by the addition of 35–37% HCl solution to DMEM containing 1,500 mg/L glucose supplemented with heat-inactivated 10% FBS by measuring pH with a FiveEasy Plus FEP20 pH Meter (METTLER TOLEDO, Tokyo, Japan). LS180 cells were grown for a sufficient time in normal medium and were subsequently maintained in the acidic cell culture medium (pH 6.5) for a few days as described previously with some modifications [26-28].

**Materials and Methods (Line 76 – 78)**

LS180 cells were cultured until 70–80% confluent in the normal (for all experiments) or the acidic medium (for isolation of total RNA, flow cytometric analysis, and cell viability assay), and then ～.

**Results (Line 304 – 355)**

Effect of siRNAs against each ERM on the expression levels of target mRNAs in LS180 cells under the acidic condition.

To confirm whether siRNAs against each ERM have similar effects on the target mRNA in the acidic microenvironmental condition, mRNA levels of each ERM were measured 3 days after transfection of LS180 cells with the siRNAs under the acidic condition with pH 6.5. siRNAs for ezrin, radixin, and moesin achieved 74%–85% reduction in the expression of their target mRNAs compared with transfection reagent alone without any significant influence on the expression of other genes (Figs 4A–C). Notably, none of the siRNAs used in this study also altered the viability of LS180 cells under the acidic condition with pH 6.5 (Fig 4D).

Fig 4. Effect of siRNAs Targeting Ezrin, Radixin, or Moesin on the mRNA Expression of Ezrin, Radixin, and Moesin (ERM) in LS180 cells under the Acidic Condition. Cells were treated with the transfection medium (Untreated), transfection reagent (Lipofectamine), nontargeting control (NC) siRNA, and specific siRNAs for ezrin, radixin, or moesin and then incubated for 3 days under the acidic condition with pH 6.5. (A–C) Expression of each mRNA in the cells treated with siRNAs relative to that in cells treated with the transfection reagent alone was measured by real-time quantitative reverse transcription-polymerase chain reaction. n = 3, **p < 0.01, *p < 0.05 vs. Lipofectamine. (D) Cell viability was assessed with the PrestoBlue cell viability reagent. Staurosporine was used as a positive control for inducing cell death. n = 6, ***p < 0.001 vs. Lipofectamine. All data are expressed as the mean ± SEM and were analyzed by one-way ANOVA followed by Tukey’s test.

Effect of ERM Silencing on the Expression of P-gp in LS180 Cells under the acidic condition.

We asked whether the gene silencing of ezrin, radixin, and moesin influence the level of ABCB1 mRNA and the expression of P-gp on the surface of the plasma membrane in LS180 cells under the acidic condition. None of these siRNAs had any effect on the levels of ABCB1 mRNA, whereas the siRNA against ABCB1 significantly decreased the levels of ABCB1 mRNA, compared to that in cells treated with the transfection reagent alone (Fig 5A). The results of flow cytometric analysis showed that silencing of ezrin, but not of radixin and moesin, significantly decreased the expression of P-gp on the surface of the plasma membrane to the same level as did the silencing of ABCB1 (Figs 5B, C).

Fig 5. Effect of siRNAs Targeting Ezrin, Radixin, or Moesin on the ABCB1 mRNA and P-gp Expression on the Surface of LS180 cells under the Acidic Condition. Cells were treated with the transfection medium (Untreated), transfection reagent (Lipofectamine), nontargeting control (NC) siRNA, and specific siRNAs for ABCB1, ezrin, radixin, or moesin and then incubated for 3 days under the acidic condition with pH 6.5. (A) The expression of ABCB1 mRNA in cells treated with each siRNA relative to that in cells treated with the transfection reagent alone was determined by real-time quantitative reverse transcription-polymerase chain reaction. n = 3, ***p < 0.001 vs. Lipofectamine. (B) An overlay of the representative histograms for the expression peak of Alexa Fluor 488-labeled P-gp on the surface of the plasma membrane of LS180 cells treated with the transfection medium (Untreated; black line), transfection reagent (Lipofectamine; gray line), ABCB1 siRNA (purple line), and ezrin siRNA (pink line), as measured by flow cytometry. The calculated relative mean fluorescence intensity peaks of P-gp on the surface of the plasma membrane for all the treatments are shown in (C). n = 4, **p < 0.01, *p < 0.05 vs. Lipofectamine. All data are expressed as the mean ± SEM and were analyzed by a one-way ANOVA followed by Tukey’s test.

**Figure 4 and 5** (See attached file named Fig 4 and Fig 5)

**Discussion (Line 453 – 466)**

Another importance is that gene silencing of ezrin, but not of radixin and moesin significantly decreased the cell surface expression of P-gp without affecting the levels of the ABCB1 mRNA in LS180 cells cultured at the acidic pH condition which mimics the actual tumor microenvironment. These results are in good agreement with those observed in the normal culture condition. The extracellular acidity is a common hallmark of almost all tumors due to the accumulation of lactic acid and H+, leading to the microenvironmental acidification [28, 68, 69]. Previous studies have shown that microenvironmental acidic pH exerts divergent roles in tumor development such as invasion, dissemination, and metastasis [70-72]. Moreover, a growing body of recent evidence suggests that acquired MDR not only to a conventional chemotherapeutics but also to molecular targeted drugs can also be caused by the highly acidic microenvironment of tumors [69]. Taken together, ezrin may play a key role in the plasma membrane localization of P-gp at the acidic tumor microenvironment, leading to an acquired MDR in a variety of cancer cells.

**References (Line 610 – 622, and Line 788 – 806)**

26.	Logozzi M, Angelini DF, Iessi E, Mizzoni D, Di Raimo R, Federici C, et al. Increased PSA expression on prostate cancer exosomes in in vitro condition and in cancer patients. Cancer Lett. 2017;403:318-29. Epub 2017/07/12. doi: 10.1016/j.canlet.2017.06.036. PubMed PMID: 28694142.

27.	Logozzi M, Mizzoni D, Angelini DF, Di Raimo R, Falchi M, Battistini L, et al. Microenvironmental pH and Exosome Levels Interplay in Human Cancer Cell Lines of Different Histotypes. Cancers (Basel). 2018;10(10). Epub 2018/10/12. doi: 10.3390/cancers10100370. PubMed PMID: 30301144.

28.	Boussadia Z, Lamberti J, Mattei F, Pizzi E, Puglisi R, Zanetti C, et al. Acidic microenvironment plays a key role in human melanoma progression through a sustained exosome mediated transfer of clinically relevant metastatic molecules. J Exp Clin Cancer Res. 2018;37(1):245. Epub 2018/10/07. doi: 10.1186/s13046-018-0915-z. PubMed PMID: 30290833.

68.	Cairns RA, Harris IS, Mak TW. Regulation of cancer cell metabolism. Nat Rev Cancer. 2011;11(2):85-95. Epub 2011/01/25. doi: 10.1038/nrc2981. PubMed PMID: 21258394.

69.	Pillai SR, Damaghi M, Marunaka Y, Spugnini EP, Fais S, Gillies RJ. Causes, consequences, and therapy of tumors acidosis. Cancer Metastasis Rev. 2019;38(1-2):205-22. Epub 2019/03/27. doi: 10.1007/s10555-019-09792-7. PubMed PMID: 30911978.

70.	Robey IF, Baggett BK, Kirkpatrick ND, Roe DJ, Dosescu J, Sloane BF, et al. Bicarbonate increases tumor pH and inhibits spontaneous metastases. Cancer Res. 2009;69(6):2260-8. Epub 2009/03/12. doi: 10.1158/0008-5472.CAN-07-5575. PubMed PMID: 19276390.

71.	Estrella V, Chen T, Lloyd M, Wojtkowiak J, Cornnell HH, Ibrahim-Hashim A, et al. Acidity generated by the tumor microenvironment drives local invasion. Cancer Res. 2013;73(5):1524-35. Epub 2013/01/05. doi: 10.1158/0008-5472.CAN-12-2796. PubMed PMID: 23288510.

72.	Rofstad EK, Mathiesen B, Kindem K, Galappathi K. Acidic extracellular pH promotes experimental metastasis of human melanoma cells in athymic nude mice. Cancer Res. 2006;66(13):6699-707. Epub 2006/07/05. doi: 10.1158/0008-5472.CAN-06-0983. PubMed PMID: 16818644.

**Reply comments to #Reviewer 2**

We would like to thank #Reviewer 2 for constructive opinion and evaluation on our manuscript. We have carefully read all of your comments and suggestions and have incorporated them into the revised version of manuscript. Detailed responses to your comments are listed below, and we highlighted all changes with word track changes in the file labeled ‘Revised Manuscript with Track Changes’. We hope this revised manuscript would be satisfactory for publication in PLoS One.

***Comment 1, line 233, the the***

**Reply Comments.**

As #Reviewer 2 pointed out, we corrected a typo. Thank you for careful reading.

***Comment 2, The Figures are in low resolution that were hard to identify.***

**Reply Comments.**

We are sorry it is a bit hard to see the figure in the PDF file because figure resolutions are very low. Therefore, we revised all Figures fallen within the size, dimensions, and resolution using PACE assistance system according to the instruction of this journal as far as possible in the revised version.

***Comment 3, line 301, would it be too high concentration of 50 μM of verapamil?***

**Reply Comments.**

With respect to the concentration of verapamil used in this study, 50 μM of verapamil is often used in the *in vitro* experiments to inhibit the transport activity of P-gp in many previous studies [1-6]. However, as #Reviewer 2 pointed out, we think that 50 μM of verapamil seems to be a relatively high concentration after careful consideration. Therefore, we performed additional experiments to determine whether lower concentration of verapamil also inhibits the transport activity of P-gp, leading to the accumulation of Rho123, a typical fluorescence substrate for P-gp, in LS180 cells by confocal laser scanning microscopy analysis. Based on the results of additional experiments, we added following sentence and figure in the Results and Supporting Information (S1 Fig. and S1 File Supplemental materials and methods) to the revised version of manuscript.

**References for this comment**

1.	Jodoin J, Demeule M, Beliveau R. Inhibition of the multidrug resistance P-glycoprotein activity by green tea polyphenols. Biochim Biophys Acta. 2002;1542(1-3):149-59. Epub 2002/02/21. doi: 10.1016/s0167-4889(01)00175-6. PubMed PMID: 11853888.

2.	Rigalli JP, Ruiz ML, Perdomo VG, Villanueva SS, Mottino AD, Catania VA. Pregnane X receptor mediates the induction of P-glycoprotein by spironolactone in HepG2 cells. Toxicology. 2011;285(1-2):18-24. Epub 2011/04/05. doi: 10.1016/j.tox.2011.03.015. PubMed PMID: 21459122.

3.	Shang H, Wang Z, Ma H, Sun Y, Ci X, Gu Y, et al. Influence of verapamil on the pharmacokinetics of rotundic acid in rats and its potential mechanism. Pharm Biol. 2021;59(1):200-8. Epub 2021/02/18. doi: 10.1080/13880209.2021.1871634. PubMed PMID: 33595422.

4.	Zhao L, Zhao Y, Schwarz B, Mysliwietz J, Hartig R, Camaj P, et al. Verapamil inhibits tumor progression of chemotherapy-resistant pancreatic cancer side population cells. Int J Oncol. 2016;49(1):99-110. Epub 2016/05/14. doi: 10.3892/ijo.2016.3512. PubMed PMID: 27177126.

5.	Zhou Y, Song X, Dong G. Effects of verapamil on the pharmacokinetics of puerarin in rats. Xenobiotica. 2019;49(10):1178-82. Epub 2018/09/04. doi: 10.1080/00498254.2018.1518552. PubMed PMID: 30173622.

6.	Zhang Y, Li J, Lei X, Zhang T, Liu G, Yang M, et al. Influence of Verapamil on Pharmacokinetics of Triptolide in Rats. Eur J Drug Metab Pharmacokinet. 2016;41(4):449-56. Epub 2015/04/10. doi: 10.1007/s13318-015-0275-4. PubMed PMID: 25854676.

**Results (Line 371 – 373)**

Of note, we validated that treatment of LS180 cell with 5, 10, and 50 μM of verapamil dose-dependently increased the uptake capacity of Rho123 (S1 Fig.).

**Supporting information** (an attached PDF file named S1_fig.)

**Supplemental materials and methods**

**Rho123 accumulation study**

LS180 cells were seeded at a density of 1.0 × 10^5^ cells/dish in polylysine-coated 35-mm glass bottom dish with 14 mm inner diameter (Matsunami Glass, Osaka, Japan) and were incubated overnight at 37°C in a humidified atmosphere with 5% CO_2_ to allow for attachment. Then cells were pretreated with 5, 10 or 50 μM of verapamil (FUJIFILM Wako Pure Chemical, Osaka, Japan), a classical inhibitor of the P-gp function, for 30 min followed by incubation with 10 μM of Rhodamine123 (Merck, Darmstadt, Germany) as a P-gp substrate in the dark for 2 h at 37°C in a humidified atmosphere with 5% CO_2_. After washing the cells with D-PBS for three times, photomicrographs were taken at 0.5–1.0-μm intervals for z-axis at an original magnification of 20× with a Nikon A1 confocal laser microscope system (Nikon Instruments, Tokyo, Japan). The two- or three-dimensional images were reconstructed from the obtained pictures using the NIS-Elements Ar Analysis software (Nikon Instruments).

**Supplemental figure**

**S1 Fig. Intracellular Accumulation of Rhodamine123 in LS180 Cells after Treatment with Verapamil in a Concentration-dependent Manner.** Cells were treated with verapamil at a concentration of 5, 10, and 50 μM and then incubated for 30 min followed by incubation with 10 μM of Rhodamine (Rho) 123. Representative confocal laser scanning microscopy images of fluorescent Rho123 and of each overlay with phase contrast in LS180 cells. Upper and lower panels indicate Rho123 and merge of Rho123 with phase contrast image, respectively. Scale bars: 50 μm. All the images are representative of at least three independent experiments.

***Comment 4, ABCB1 mRNA vs. P-gp mRNA, they are inconsistent in Results and Discussion (line 373)*
Reply Comments.**

As #Reviewer 2 pointed out, we corrected P-gp mRNA into a suitable expression, i.e., ABCB1 mRNA and used the same terms throughout the document. Thank you for careful reading.
